# Investigating the Potential of Generative AI Clinical Case-Based Simulations on Radiography Education: A Pilot Study

**DOI:** 10.1007/s10278-025-01601-8

**Published:** 2025-07-08

**Authors:** Da Zhong, Steven Kwok Keung Chow

**Affiliations:** 1 DaZhou Vocational and Technical College, DaZhou, China; 2 Clinical Research and Imaging Centre, South Australian Health and Medical Research Institute, Adelaide, SA 5000 Australia; 3 Department of Medical Imaging and Radiation Sciences, Faculty of Medicine Nursing and Health Sciences, School of Primary and Allied Health Care, Monash University, Wellington Road, Clayton, Melbourne, VIC 3800 Australia

**Keywords:** Medical imaging technologists, Radiography, Information and communications technology (ICT) in education, Generative artificial intelligence, Online learning, Diagnostic imaging

## Abstract

Education for medical imaging technologists or radiographers in regional and rural areas often faces significant challenges due to limited financial, technological, and teaching resources. Generative AI presents a promising solution to overcome these barriers and support the professional development of radiographers. This pilot study aimed to evaluate the educational value of an in-house AI-based imaging simulation tool designed to generate clinically relevant medical images for professional training purposes. In July 2023, a professional development lecture featuring AI-generated clinical imaging content was delivered to students (*N* = 122/130) and recent graduates (*N* = 155/532), alongside a pre-lecture survey. Following the session, participants completed a questionnaire comprising structured and open-ended items to assess their understanding, perceptions, and interest in AI within medical imaging education. Survey results indicated that both students and graduates possessed a foundational awareness of AI applications in medical imaging. Graduates demonstrated significantly higher expectations for clinical realism in AI-generated simulations, likely reflecting their clinical experience. Although the simulator’s current capabilities are limited in replicating complex diagnostic imaging, participants acknowledged its pedagogical value, particularly in supporting basic anatomical education. Approximately 50% of respondents expressed interest in further developing their AI knowledge and contributing to the research and development of AI-based educational tools. AI-driven imaging simulation tools have the potential to enhance radiography education and reduce teaching barriers. While further development is needed to improve clinical fidelity, such tools can play a valuable role in foundational training and foster learner engagement in AI innovation.

## Introduction

In recent years, the development of artificial intelligence (AI) has significantly influenced teaching methods in the education of medical imaging technologists (MITs) and radiographers [1–3]. AI has the potential to enhance interactivity and enrich the learning experience by supporting personalized and adaptive instruction [4]. For example, AI can simulate teacher responses [5] and generate question banks to improve the accuracy and efficiency of assessments [6]. Furthermore, AI algorithms are capable of analyzing large datasets, including medical records and diagnostic images, to provide insights into potential diagnoses and treatment options [7, 8]. This technological innovation offers a promising tool for assisting medical professionals in delivering accurate and timely diagnostic information, particularly in complex cases where traditional methods may be limited [3, 9, 10]. As AI continues to advance, there is growing interest among medical imaging professionals in adopting these innovations through continuing professional development (CPD) programs and/or specialized workshops [11]. In response to these trends, the education and training of radiographers are evolving to align with the demands of modern healthcare systems [12]. Therefore, it is essential for radiography professionals to be trained not only in the operation of AI technologies but also in integrating AI tools into clinical workflows and interpreting AI-generated outputs [2, 13, 14]. With appropriate training, medical imaging professionals will be better equipped to adopt AI technology, leading to improved diagnostic accuracy and enhanced patient outcomes.

### Radiography Education

Radiography professionals are required to demonstrate a high level of proficiency and adaptability in performing medical diagnostic procedures [15]. Radiographers play a vital role in delivering accurate and timely diagnostic information that supports clinical decision-making [15]. However, traditional training methods often fall short in fully preparing students for the wide range of imaging scenarios they may encounter in clinical practice [1]. Radiography education typically combines classroom-based theoretical instruction with hospital-based clinical placements to ensure students acquire both foundational knowledge and hands-on experience [16]. One of the ongoing challenges in radiography training is keeping pace with the rapid development of advanced imaging modalities, including X-ray, magnetic resonance imaging (MRI), and computed tomography (CT) [17]. As medical technologies evolve, radiography professionals must continually update their skills and knowledge to remain competent in a dynamic clinical environment [18]. Furthermore, the availability of advanced imaging equipment and experienced educators varies considerably between regions, contributing to disparities in the quality of education and clinical exposure [19, 20]. These challenges are particularly pronounced in regional areas of China, where longstanding limitations in educational infrastructure continue to hinder the development of a uniformly trained radiography workforce.

Another significant challenge facing radiography education in regional areas is the difficulty of keeping pace with rapidly evolving technologies, particularly the integration of artificial intelligence (AI) in medical imaging [14]. While institutions in urban centers have begun to incorporate AI tools and digital workflow training into their curricula, regional colleges often fall behind due to constraints such as limited funding, outdated infrastructure, and fewer collaborations with industry stakeholders [19, 21]. As a result, graduates from these regions may have limited exposure to AI applications, which can affect their preparedness for roles within modern, technology-driven healthcare environments [22]. Integrating AI into both clinical imaging practice and radiography education holds promise as a means of bridging this gap.

### Use of AI in Medical Imaging

The integration of generative AI into clinical training for radiographers’ education could be a pioneering approach to radiographer education [1, 23]. Tools such as ChatGPT provide a viable solution for this quandary, offering a virtual tutor or generating clinical case studies to support radiography education [5, 24]. The virtual environment provided by radiography education accurately recreates complex clinical scenarios [5]. One potential solution is to develop ChatGPT, an online tool that simulates typical clinical case studies to evaluate radiographers’ clinical judgment and decision-making abilities [24]. Accurate evaluations of diagnostic accuracy, sensitivity, specificity, and justification of imaging examinations are crucial for assessing the performance of medical imaging professionals [1, 5]. This approach could provide guidelines for real-world scenarios, thus enabling a more accurate evaluation of the proficiency of professionals [5].

In the learning process, the simulations could increase the complexity, presenting challenging cases that require the application of real-world diagnostic skills and knowledge to improve quick decision-making [23]. By enhancing the fairness and adaptability of generative AI tools, it is possible to develop customized and comprehensive training programs tailored to individual learning needs within radiography education [4]. Using medical imaging simulations, students can participate in immersive and interactive learning experiences that closely resemble real-world scenarios and challenges they may encounter in a clinical setting [25]. Moreover, generative AI could have strong potential in describing medical terminology and human anatomical structures [5]. This innovative approach could enhance educational experiences and cultivate highly critical thinking skills in clinical training programs [3, 11, 26].

Generative AI could have potential advantages for the medical imaging education of radiographers. However, the perceptions of radiography students and professionals should be investigated in regional areas of China. This study examines the potential benefits, challenges, and implications of incorporating AI-driven simulations into educational curricula. The aims of this study are:

1. To investigate the perceptions of radiography students and professionals in regional areas of China regarding the use of AI and Generative AI tools in the workplace or education.
2. To develop and integrate a generative AI simulation tool to produce educational clinical case scenarios for use in radiography training.
3. To investigate the potential of generative AI in simulating realistic clinical cases for radiography education.

## Method

This study was conducted at one vocational and technical college, a tertiary-level institution located in a regional area of China. The primary objective of the research was to explore insights from radiography graduates, current students, and lecturers regarding the implementation of generative AI in radiography education, with a particular focus on regional contexts in China. An anonymous electronic survey was distributed via email to radiography graduates from the past ten years, using contact details obtained from the college's registrar database. A total of 532 surveys were sent to the radiography graduates, with no follow-up reminders issued. The online survey was made available to students and graduates for a two-week period between October and December 2023. A total of 12 questions were distributed through the institution's online learning platform. The survey covered sections on background information, including sex and trained imaging modality, knowledge of AI in medical imaging or radiotherapy, perceptions and understanding of AI among radiographers, and perceived challenges in integrating AI.

In the second phase of the study, a mini-application for generating medical images was developed to facilitate the fusion of medical image data. Eight lectures incorporating clinically relevant images generated by this application were delivered to current students. The lecturers documented their observations on the image generation process and collected feedback from students during the lecture. Additionally, lecture recordings and supplementary notes were made available to graduates through the institution’s online learning platform. To support further development of the mini-application, questionnaire responses from both students and graduates were collected and analysed. The questionnaires were distributed via the same platform following the lecture, consisting of 12 questions and an open-ended comment section. These questionnaires focused on participants’ perceptions of clinical case imaging, their knowledge of AI, and the perceived challenges and future directions for AI integration in the field. The same questionnaires were administered to graduates, lecturers, and current students to ensure consistency in data collection across participant groups. All survey and questionnaire materials were translated into English.

### Survey and Questionnaire Design

Initially, the survey and questionnaire were distributed to graduates, current students, and lecturers of the radiography program at the vocational and technical college. The survey and questionnaire were developed by the first author and subsequently reviewed by the second author to ensure alignment with the local context and content. A total of eight lecturers from the institution also participated by completing the survey and questionnaire. Participants were informed through a cover letter about the public and shared nature of the study data. In accordance with ethical guidelines, all responses were fully anonymized, and no withdrawal mechanism was provided. Informed consent was obtained from all participants. The survey and questionnaire were designed and structured according to established research methodology standards [27, 28]. A 5-point scale design was employed for the survey and questionnaire [27].

### Mini-Applications

The mini-application was developed using the Imagine API (http://www.imagine.art). The API was developed using Python 3.7 on a MacBook Pro (model no. 2141) with an Intel Core i9 CPU and 16 GB of memory. The OpenAI API library DALL·E 3 was used to generate or manipulate images. The API can perform medical image fusion without MR or CT images. The clinical case images were generated without any source images for fusion.

### Statistical Analysis

A statistical analysis was performed to investigate any significant relationship between the results and the perception and visibility of adopting generative AI case simulations in radiography education. All the statistical analyses were performed using SPSS 16.0 (Chicago, IL). All the statistical tests were two-sided, and the means and standard deviations (SD) were used. A *p*-value of 0.05 was considered to indicate statistical significance.

## Results

A total of 122 out of 130 students participated in the survey, comprising 42 first-year, 39 s-year, and 41 third-year students. Among the participants, 69 were female and 53 were male, with ages ranging from 24 to 26 years and a mean age of 24.5 years. Additionally, five radiography lecturers from the study’s vocational and technical college participated in the study. The mean age of lecturers was 42.3 years, with a gender distribution of three males and two females. Furthermore, 152 out of 532 radiography graduates responded to the questionnaire (*N* = 152; 79 females and 73 males). Detailed demographic information is presented in Table 1.

**Table 1:** Background information on the radiography graduates who participated in the survey

	Male (*N* = 73)		Female (*N* = 79)		*P*-value
	*N*	%	*N*	%	
Specialization (can select more than one)					
X-ray	66	90.41	59	74.68	0.252
CT	45	61.64	43	54.43	0.09
MRI	53	72.26	33	41.77	< 0.001*
Do you know about AI in medical imaging?					
X-ray:					
Yes	20	37.7	13	32.5	0.027*
No	12	60	18	46.1	
CT and MRI:					
Yes	33	62.3	27	67.5	
No	8	40	21	53.9	
Will you attend AI lectures or in-house training?					
X-ray:					
Yes	11	29.7	10	28.6	0.5
No	17	68	15	51.7	
No comment	5	45.5	5	38.5	
CT and MRI:					
Yes	26	70.3	25	71.4	
No	8	32	14	48.3	
No comment	6	54.5	8	61.5	
Do you use AI tool in medical imaging?					
X-ray:					
Yes	11	40.7	12	48	0.387
No	23	50	23	42.6	
CT and MRI:					
Yes	16	59.3	13	52	
No	23	50	31	57.4	
Does your workplace have AI integration plan?					
X-ray:					
Yes	5	38.5	12	48	0.757
No	32	53.3	21	38.2	
CT and MRI:					
Yes	8	51.5	13	52	
No	28	46.7	34	61.8	
Does your radiologist encourage AI integration?					
X-ray:					0.148
Yes	18	48.6	20	60.6	
No	18	50	19	41.3	
CT and MRI:					
Yes	19	51.4	13	39.4	
No	18	50	27	58.7	
What type of leaning format on AI training?					
Web seminar	33	45.21	39	49.37	0.015*
Continued professional education (CPD)	15	20.55	27	34.18	
Research articles	25	34.25	13	16.45	
Reservations about AI					
Increase the workload	15	20.55	21	26.58	0.007*
Complaints by patient	13	17.8	18	22.78	
Future career	24	32.88	22	27.84	
Problem working with radiologist	12	16.44	13	16.46	
No reservation	9	12.33	5	6.34	

*P-values < 0.05 were calculated by Chi-square χ^2^ test

### Background Information on AI Knowledge of Radiography Graduates

Table 1 presents demographic information on radiography graduates’ knowledge of AI in medical imaging based on survey responses. The results indicate that most respondents, 72.60% of male graduates and 50.63% of female graduates, were aware of AI applications in medical imaging. However, over 60% of graduates reported lacking practical experience with AI tools, and no established plans for AI implementation existed in their workplaces. Approximately 60% of graduates acquired AI knowledge through training sessions, webinars, and CPD, while only around 30% gained information from research articles. Despite this exposure, most graduates had not received formal workplace training on AI use, with 50% expressing interest in attending lectures or in-house training programs. Opinions regarding AI integration varied among radiologists in the workplace. Nevertheless, the majority of graduates recognized the potential of AI in medical imaging, particularly in areas such as body region segmentation, image quality enhancement, and radiography education.

### Background Information AI Knowledge of Radiography Students

**Fig. 1:**
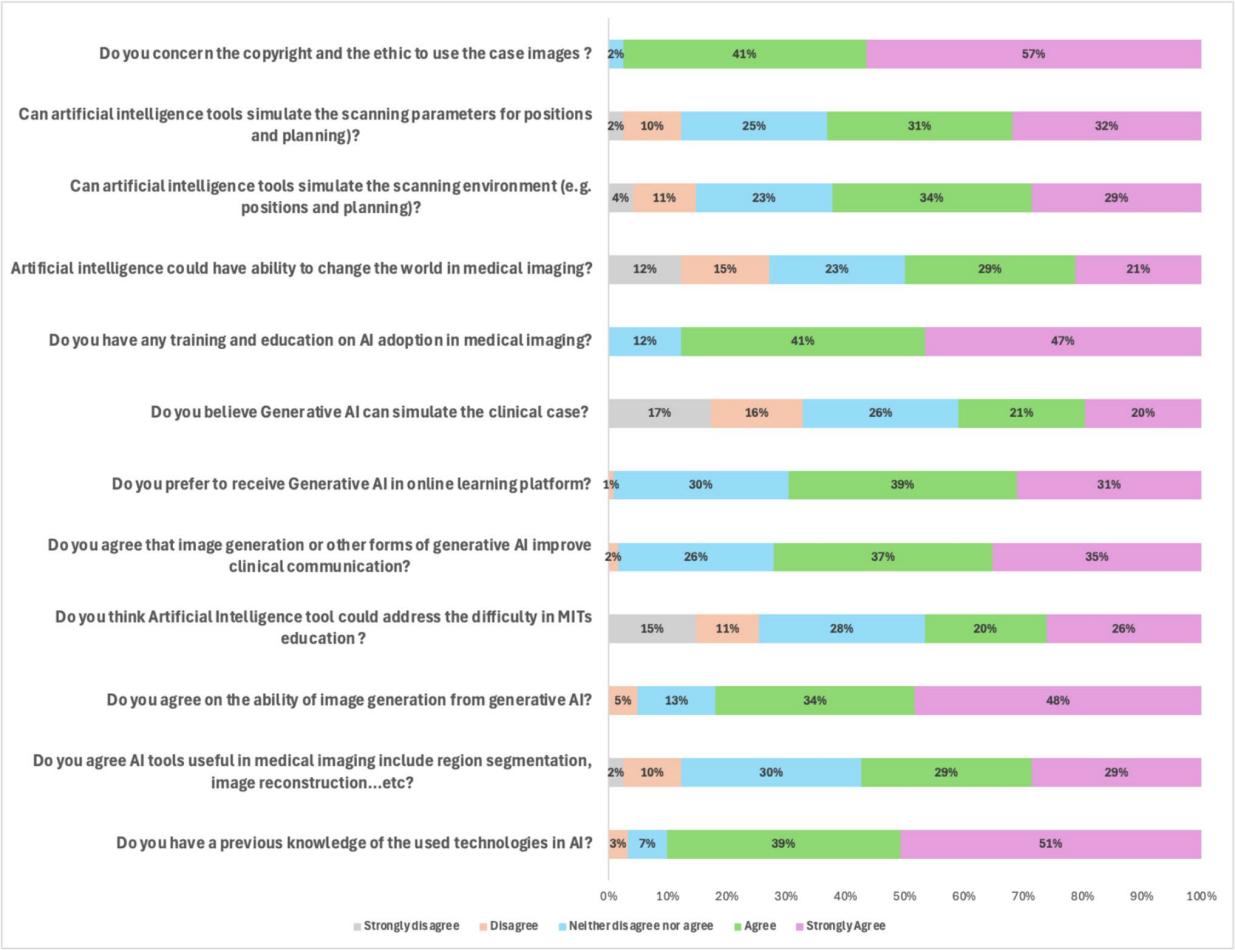
Survey results (*n* = 122) on students’ perceptions and understanding of generative AI in radiography education

The results of a survey conducted among radiography students regarding their perception and understanding of generative AI within the educational program are presented in Fig. 1. The survey indicated that most students possess the necessary knowledge and training to operate AI technology, particularly in the field of medical imaging. Specifically, 63% of students believed that AI tools could effectively simulate critical aspects such as scanning parameters, scanning positions, and patient planning. Furthermore, 99% agreed that AI can be useful for cancer or body region segmentation and image reconstruction in medical imaging. However, only 50% of students believed that AI has the potential to transform its role in medical imaging and enhance clinical communication through various forms of generative AI. The survey also revealed a split in confidence regarding AI’s ability to alleviate study difficulties. Moreover, half of the students were doubtful, while the other half considered AI a significant aid. Despite most students having received training in AI applications in medical imaging, many expressed interest in integrating generative AI into online learning platforms to enhance their practical experience. Finally, copyright and ethical concerns related to the use of case images in radiography education were raised by both students and graduates.

### Feedback From Lecturers on AI in Medical Imaging Lectures

**Fig. 2:**
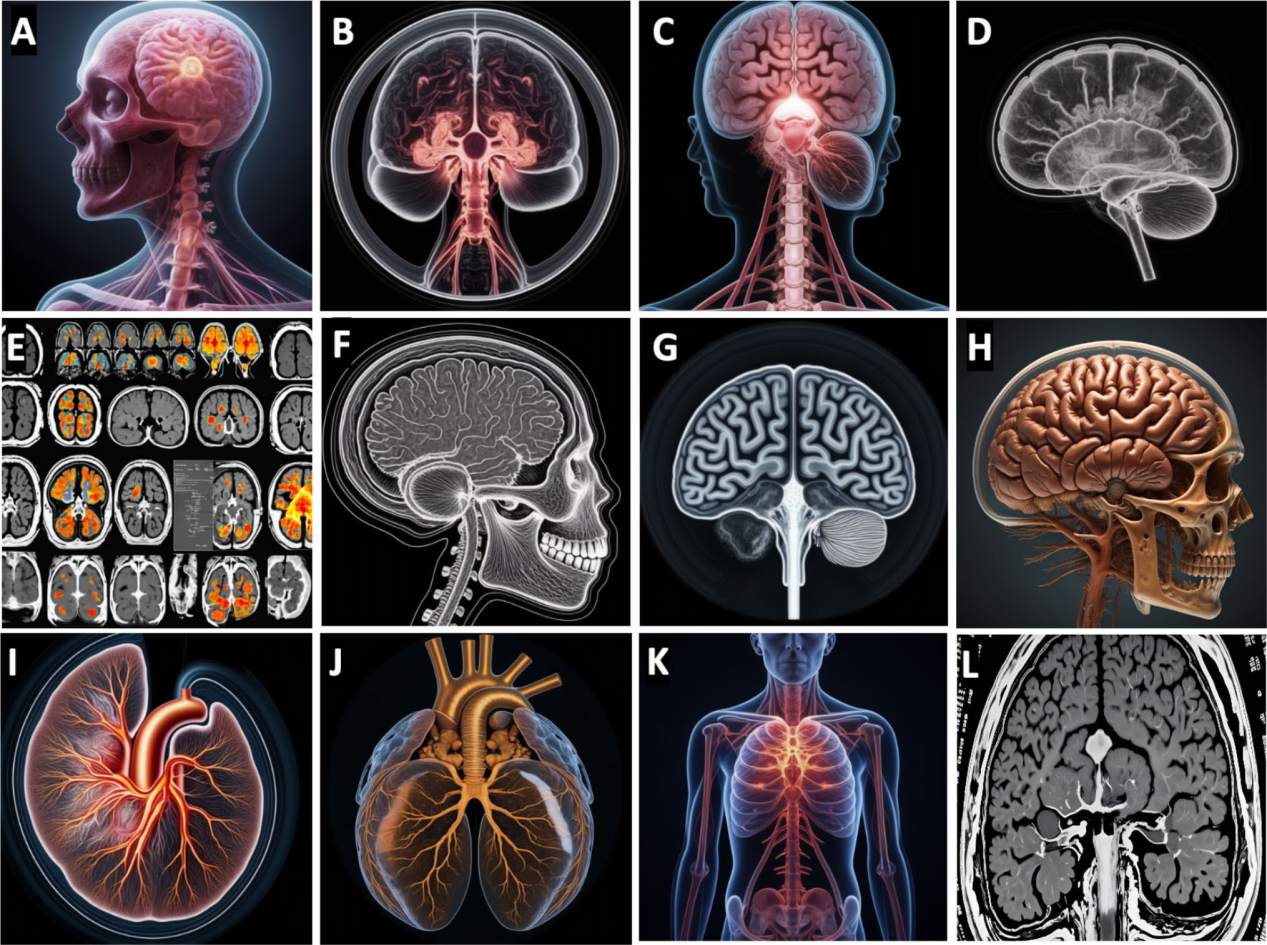
Generative AI simulator tool developed in-house that generated brain cancer and cardiac simulated images for AI in medical imaging lecture presentations using the following keywords: brain cancer and cardiac MRI or CT modalities

Figure 2 displays images generated by our proprietary generative AI simulator tool during a medical imaging lecture. A short video created by the in-house-developed generative AI simulator, focusing on brain cancer in MR images, is included in the supplemental data. The images shown in Fig. 2A through D and E through H correspond to “human brain cancer” in medical and MRI imaging, respectively. Figures I through K were generated using the keyword “human cardiac medical imaging,” while Fig. 2L was based on the keyword “brain cancer in MRI or CT imaging.” The lecturers found Fig. 2A to E to be particularly representative of brain cancer and decided to incorporate them into the radiography education curriculum. However, they did not approve the use of Fig. 2F to L. Nevertheless, the lecturers welcomed the use of the images from Fig. 2 in introductory or anatomy courses to enhance students’ understanding of the subject matter. They also noted that the simulator’s capabilities extend beyond basic medical imaging keywords. Finally, the lecturers unanimously agreed that the generative AI simulator requires further training, testing, and validation before full integration into radiography education. They believe that AI should be introduced and/or integrated into radiography programs to raise awareness and promote further research in this field. As AI technology matures, simulators could be embedded into online learning platforms as individual assessment tools for radiography students.

### The Questionnaire Results After the Lectures on Students and Graduates

**Fig. 3:**
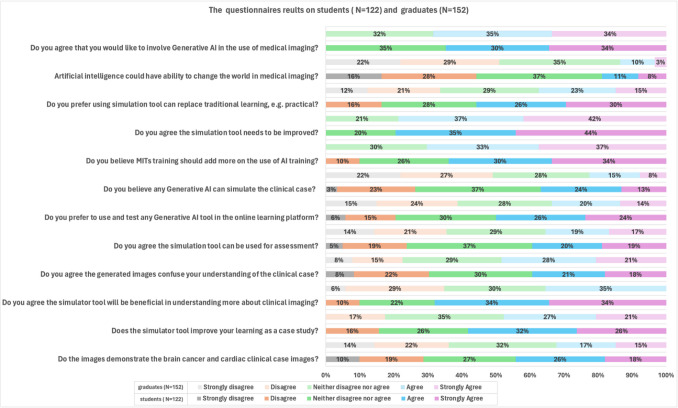
Questionnaire results for students (*n* = 122) and graduates (*n* = 155) after an AI lecture on medical images

The lectures on medical imaging AI were delivered to students and graduates through web-based instruction, followed by a questionnaire that included open-ended questions. Figure 3 shows the questionnaire results for students and graduates after an AI lecture on medical imagesnd. Table 2 shows the statistical results on the questionnaire results on students’ perceptions and understanding of generative AI in radiography education. The questionnaire results indicated that 32% of graduates and 44% of students reported having clinical experience with cases related to brain and cardiac cancers. Additionally, 48% of graduates and 58% of students believed that AI tools could enhance case-based clinical lessons. Notably, there was a significant 34% difference in students’ perceptions regarding the usefulness of the AI tool compared to graduates. After the lecture, 10% more graduates than students found the generated images confusing. In contrast, 14% more students than graduates perceived the AI tool to be more effective when used via the online learning platform. These differences may be attributed to variations in clinical experience and training in medical imaging practice.

**Table 2:** Chi-square (*χ*^2^) test results based on questionnaire data (*n* = 122) examining students’ perceptions and understanding of generative AI in radiography education

Questions	Chi-square *χ*^2^ statistic	*P*-value	Significance
Do you have a previous knowledge of the used technologies in AI?	133.25	< 0.00001	Significant
Do you agree AI tools useful in medical imaging include region segmentation, image reconstruction, etc.?	40.79	< 0.00001	Significant
Do you agree on the ability of image generation from generative AI?	101.52	< 0.00001	Significant
Do you think artificial intelligence tool could address the difficulty in MITs education?	13.16	0.0105	Significant
Do you agree that image generation or other forms of generative AI improve clinical communication?	78.9	< 0.00001	Significant
Do you prefer to receive generative AI in online learning platform?	80.87	< 0.00001	Significant
Do you believe generative AI can simulate the clinical case?	**4.15**	0.38641*	**Not significant**
Do you have any training and education on AI adoption in medical imaging?	122.84	< 0.00001	Significant
Artificial intelligence could have ability to change the world in medical imaging?	10.54	0.03224	Significant
Can artificial intelligence tools simulate the scanning environment (e.g. positions and planning)?	37.18	< 0.00001	Significant
Can artificial intelligence tools simulate the scanning parameters for positions and planning)?	42.67	< 0.00001	Significant
Do you concern the copyright and the ethic to use the case images?	175.95	< 0.00001	Significant

Furthermore, graduates showed a stronger interest in supplementing their studies with additional information on the application of AI in radiography research, whereas only 10% of students felt that the current information was sufficient. A large majority, 80% of both students and graduates, agreed that the AI simulator requires further improvement. Students expressed greater enthusiasm for using clinical simulation tools as substitutes for traditional practical training compared to graduates, with an approximate 20% difference. Both groups believe that AI holds the potential to enhance ICT in radiography education. Finally, both students and graduates expressed a desire to actively participate in evaluating generative AI within the context of medical imaging by comparing study outcomes.

### Results on the Open-Ended Questions

The results of the open-ended questions from students and graduates after the clinical case on the AI simulation lecture: Only 20% of graduates (*N* = 35) and students (*N* = 32) provided their comments on the after-lecture questionnaire. The comments were mostly similar ideas. The open-ended comments were categorized into major positive and negative comments. The results expressed a variety of opinions, reflecting both support for and concerns about the integration of AI-based simulation tools in radiography education (Table 3). In the following section, negative comments are presented in bold for emphasis, while the comments without bold reflect positive perspectives. The comments were collected from both students and graduates:

- **1. I am interested in the ChatGPT tool and how it can be used in radiography education.**
- **2. The keywords used in the clinical case should be more detailed to provide a clearer understanding.**
- 3. The accuracy of the clinical case can be improved, especially when interpreting challenging images.
- **4. The image provided does not seem to be a realistic demonstration of a real-life situation in a brain cancer scenario.**
- 5. The AI simulator would benefit from additional training datasets or other deep learning models.
- 6. This tool could be fully automated and integrated into scanning consultations for improved efficiency.
- 7. As a limited resource in the classroom, this tool could be highly helpful in teaching anatomy.
- **8. This tool could be more effectively used for teaching anatomy than for case study training.**
- **9. AI could be used for segmentation but may not be suitable for teaching purposes.**
- 10. This tool could be used in year one teaching and online assessment tools to identify body regions rather than specific case studies.
- **11. AI image generation may not be suitable for clinical cases and is generally a broad tool.**
- 12. Radiography education is a challenging field that involves human interaction and expertise. AI cannot fully replace human input.

**Table 3:** Chi-square (*χ*^2^) test results on the questionnaire results for students (*n* = 122) and graduates (*n* = 155) after an AI lecture on medical images

Questionnaire question	Student *χ*^2^	Student *p*-value	Student significance	Teacher *χ*^2^	Teacher *p*-value	Teacher significance
Do the images demonstrate the brain cancer and cardiac clinical case images?	12.02	0.01723	Significant	15.94	0.00311	Significant
Does the simulator tool improve your learning as a case study?	**6.85**	**0.07675**	**Not significant**	11.01	0.01166	Significant
Do you agree the simulator tool will be beneficial in understanding more about clinical imaging?	19.57	0.00021	Significant	32.02	< 0.00001	Significant
Do you agree the generated images confuse your understanding of the clinical case?	15.62	0.00357	Significant	24.71	0.00006	Significant
Do you agree the simulation tool can be used for assessment?	31.44	< 0.00001	Significant	10.00	0.04043	Significant
Do you prefer to use and test any Generative AI tool in the online learning platform?	22.84	0.00014	Significant	11.10	0.02550	Significant
Do you believe any Generative AI can simulate the clinical case?	38.74	< 0.00001	Significant	23.35	0.00011	Significant
Do you believe MITs training should add more on the use of AI training?	16.30	0.00099	Significant	53.54	< 0.00001	Significant
Do you agree the simulation tool needs to be improved?	10.54	0.00514	Significant	10.74	0.00466	Significant
Do you prefer using simulation tool can replace traditional learning, e.g., practical?	36.85	< 0.00001	Significant	13.68	0.00840	Significant
Artificial intelligence could have ability to change the world in medical imaging?	35.79	< 0.00001	Significant	54.26	< 0.00001	Significant
Do you agree that you would like to involve Generative AI in the use of medical imaging?	**0.51**	**0.77562**	**Not significant**	**0.27**	**0.87405**	**Not significant**

## Discussion

The results indicate that radiography education is beginning to adopt AI tools in radiology. Both graduates and students demonstrated a satisfactory level of knowledge about AI applications in medical imaging, particularly in region segmentation. Furthermore, the findings suggest a strong interest among students and graduates to further develop their AI knowledge and skills within radiology. This highlights the potential for AI to enhance patient care and outcomes in medical imaging, while also meeting the evolving demands placed on radiography professionals [29]. Integrating AI simulation tools into the learning process could therefore support students in achieving better educational outcomes in radiography training.

Given that students and graduates had not received formal education or structured information about AI, many likely had limited or no understanding of its applications. As a result, the survey design incorporated only Yes/No response options to reduce uncertainty and enhance clarity in the data collected. While this approach has limitations, it allowed for more consistent interpretation of responses. Participants regarded the enhancement of AI simulation technology in medical imaging as a valuable contribution to radiography education [30]. The post-lecture questionnaire revealed a shared view among students and graduates that AI simulation offers significant benefits for learning. However, graduates expressed concerns regarding the current lack of realistic clinical images within AI simulation tools used for education. The results emphasized that the case images used in experiments were insufficiently advanced to replicate real-world clinical scenarios accurately [31]. This disparity likely stems from graduates’ greater clinical experience compared to students and underscores the need for more sophisticated case simulations.

Graduates recommended improving and expanding the current AI simulator, which is primarily used for teaching basic human anatomy in radiography training. Following the lecture, they acknowledged that generative AI holds promising potential to optimize the teaching process and enhance radiography education. Nevertheless, graduates also highlighted the importance of addressing issues related to the stability and reliability of generated images [31]. As a result, they expressed a desire to actively participate in the research and development of generative AI applications in radiography education, as reflected in the findings. These findings suggest that AI-based educational tools should be integrated gradually, with attention to aligning simulation fidelity with the clinical experience level of learners [31]. For early-year students, simplified case studies focusing on anatomical recognition and basic protocols may be most appropriate, while more advanced simulations could be introduced in later stages to reflect real-world complexity.

### Online Learning with the AI Imaging Simulator Tool

Integrating ChatGPT into online learning systems offers a more interactive and automated approach to radiography education, potentially enhancing the precision and effectiveness of educational outcomes [5, 24]. The in-house AI imaging simulator can generate detailed medical images based on extensive textual input; however, students with limited clinical experience may find this challenging. This AI imaging simulator could provide tailored and effective learning interactions, enabling students to acquire foundational knowledge of anatomy and medical imaging for rare diseases. The radiography education program aims to deliver proper training for healthcare professionals, particularly those in regional areas who face barriers to accessing educational institutions due to time and resource constraints [32]. Online learning could offer a viable solution for medical imaging professionals in these regions to acquire essential knowledge and skills [20]. This flexible approach allows students to participate remotely from home or the workplace, making it well-suited to their needs [20].

Moreover, graduates often seek more profound insights into the applications of AI in medical imaging technology. While the requirements of graduates and students may differ, their goals align in expanding their understanding of AI’s potential and future applications in clinical practice [33]. Such preparation is crucial, given the demand for timely decision-making and continual learning in evolving medical environments [34]. Additionally, online learning platforms facilitate knowledge sharing between lecturers and students, supporting ongoing professional development for medical imaging practitioners in regional or remote settings [19, 21]. This enables them to stay updated with the latest technologies and skills.

Finally, the findings underscore the importance of intensifying efforts to develop the in-house AI imaging simulator. Encouragingly, students and graduates demonstrate a willingness to engage in this developmental process. Involving students in the creation and refinement of such simulators may prove highly beneficial in enhancing their comprehension and practical experience within radiography education [31].

### Mismatches Between the Ability and the Realistic Performance of AI Tools

Since the release of ChatGPT, the capabilities of generative AI technology have advanced significantly. However, there is a tendency to overemphasize the current potential of AI in imaging simulation, particularly in medical imaging applications [24]. Graduates now have greater access to AI tools in the workplace, which raises their expectations for professional practice. AI simulator tools can help graduates navigate the evolving challenges and opportunities within clinical medical imaging [30].

AI technology used in medical imaging can be extended to clinical disease simulation, thereby enhancing both practical skills and theoretical knowledge [17, 29]. In contrast, students still pursuing radiography education often have more modest expectations due to their limited clinical exposure. Since students may not yet have developed practical skills or applied their knowledge in real-world scenarios, the transition from academic study to clinical practice can be daunting. Consequently, the demand for AI simulation tools may differ between students and graduates. AI simulators provide students with the opportunity for hands-on experience and exposure to realistic clinical cases, potentially bridging the gap between their expectations and reality [29, 31].

The results suggest that both students and graduates lack full confidence in the transformative impact of AI on medical imaging. This skepticism may reflect doubts about the promises made by AI proponents, as many current implementations rely on limited datasets that constrain the effectiveness of AI models. Despite the widespread adoption of machine learning and cognitive computing across various applications, concerns persist about the efficiency and reliability of AI tools. Although AI has demonstrated capabilities in performing highly technical tasks, its dependability is often questioned, especially when precise and objective outputs are required. Factors such as data quality, algorithm design, and unpredictable variables contribute to this unreliability. The significant gap between expectations of AI’s potential and the limitations posed by constrained data and models likely explains the skepticism observed among students and graduates.

A foundational understanding of human anatomy remains essential in medical imaging education [35, 36]. While there is room for improvement in AI’s ability to simulate complex clinical cases, AI simulators can significantly increase student engagement by providing opportunities to practice clinical imaging across multiple modalities [29, 31]. AI simulators could have a significant potential to reduce resource barriers in rural areas, facilitating fundamental education and practical experience for radiography students. Survey and questionnaire findings indicate a growing interest in the application of AI within continuing professional development (CPD) education [37]. This technology could transform the teaching of human anatomy, making it more effective and accessible, especially for learning about rare diseases [31, 35].

### Limitations and Future Work

Clinical databases and medical images are often inaccessible to the public, limiting their use for training machine learning models. Consequently, AI-generated images may not accurately resemble real clinical images. This lack of realism can increase the risk of producing unrealistic simulations, thereby reducing the effectiveness and relevance of AI models in both clinical and academic settings. To enhance the accuracy and reliability of AI tools in medical imaging education, access to comprehensive and diverse clinical databases is crucial.

Moreover, recent studies suggested that AI simulators could generate accurate and feasible clinical scenarios using only textual inputs [5, 24, 38]. This text-based simulation approach holds promise for future development, offering a cost-effective and efficient alternative to image-based simulations. However, more complex deep learning models and expansive clinical datasets are necessary to further advance image simulation technologies. Continued improvements in AI imaging simulation will enable ongoing exploration and innovation in this area.

For AI-driven tools to effectively address realistic medical challenges, a unified and comprehensive body of knowledge is required to cover a wide range of clinical scenarios [33, 35]. Although the proposed online learning platform is designed to function in both online and offline modes, larger offline clinical image databases are needed to enhance image generation capabilities.

One major challenge in integrating AI simulators into online learning platforms is ensuring the protection of patient information. Collaborating with open-source clinical datasets may offer a viable solution, while also addressing legal issues related to copyright infringement [39]. When incorporating APIs with large language models (LLMs), it is essential to provide clear information regarding image copyrights. Only images with proper copyright clearance should be used, or permission must be obtained from copyright holders.

While conventional clinical image simulations remain useful for teaching, AI-driven simulation tools offer greater convenience and effectiveness. Such tools can enhance student engagement and participation, providing a robust platform for knowledge acquisition and retention. This, in turn, better prepares students for advanced studies and clinical practice. Combining AI simulators with self-learning LLM models, such as ChatGPT, could create a transformative learning environment for radiography students, ultimately enhancing their practical performance [5, 24]. The ongoing development of AI technology presents a promising new approach to radiography education. Integrating AI simulators and self-learning LLM models into online learning systems could be a new approach to training radiographers, potentially enabling students to learn more effectively and thoroughly.

## Conclusion

The use of generative AI holds significant potential for educational applications in medical imaging technology and radiography. A recent pilot study explored the benefits of simulated clinical images generated by an in-house generative AI simulator. The findings revealed that students expressed a strong interest in integrating AI-driven simulation tools into their learning, while recent graduates emphasized a preference for simulation images that closely resemble real clinical cases. These insights suggest that consensus on the educational value of generative AI could substantially enhance radiographer training, particularly in resource-limited regions. While current AI technologies and image databases may not yet fully replicate the complexities of real-world clinical imaging, the advantages and potential of simulated clinical cases remain promising. Continued research using in-house generative AI simulators is warranted to further explore and refine this approach.

## Data Availability

All data presented in this paper are available upon request.
